# Long-term outcomes after Lichtenstein repair using titanium-coated mesh: A retrospective cohort study

**DOI:** 10.12669/pjms.37.1.2694

**Published:** 2021

**Authors:** Cagri Akalin

**Affiliations:** 1 Cagri Akalin, Assistant Professor, Department of General Surgery, Ordu Medical School, Ordu, Turkey

**Keywords:** Lichtenstein repair, Titanium-coated mesh, Polypropylene mesh, Recurrence, Foreign body feeling, Chronic pain

## Abstract

**Objective:**

To examine the long-term outcomes such as recurrence, foreign body feeling and chronic pain of titanium-coated mesh (TCM) versus standard polypropylene mesh (PM) after Lichtenstein repair (LR).

**Methods:**

In this retrospective cohort study, patients who underwent TCM and PM in LR were evaluated between May 2014 and January 2018 at Ordu University Training and Research Hospital in Turkey. Primary outcomes (age, gender, body mass index, smoking habits, comorbid diseases, American Society of Anesthesiologists score, hernia type, side of hernia, duration of hernia presentation and operative time) and secondary outcomes (surgical site occurence, recurrence, foreign body feeling and chronic pain) were analyzed. Patients were divided into two groups according to the mesh elected (TCM and PM); titanium group (TG) and polypropylene group (PG), respectively.

**Results:**

In this study, 221 patients were analyzed; TCM was used in 72 (32.6%) patients and PM was used in 149 (67.4%) patients. No difference was found between groups in terms of primary outcomes (*p*>0.05). In the analysis of secondary outcomes, surgical site occurence was similar in both groups (*p*>0.05). Recurrence was observed in 1.39% (n=1) of TG and 2.01% (n=3) of PG. No difference was found between groups in terms of recurrence (*p*=0.606). Foreign body feeling was observed in 15.3% (n=11) of TG and 27.5% (n=41) of PG. Chronic pain was observed in 4.2% (n=3) of TG and 12.8% (n=9) of PG. Significant differences were found between groups in terms of chronic pain and foreign body feeling (*p*=0.046 and *p*=0.044, respectively).

**Conclusion:**

The result of this study shows that in LR, TCM leads to less foreign body feeling and chronic pain than PM. However, there was no difference in terms of recurrence between these meshes.

## INTRODUCTION

Inguinal hernia repair is a frequent operation in general surgery but recurrence, chronic pain and foreign body feeling after an operation are the issues that should be resolved by surgeons.^1^,^2^ Lichtenstein repair (LR), one of the very frequently used methods, is a golden standard method in open inguinal hernia repair.^3^

Although meshes have some advantages such as high tensile strength and cost-efficiency, they also have complications such as seroma, foreign body feeling and chronic pain. New meshes are produced in order to reduce complications like this.^4^ One of these meshes which is made for this, titanium-coated mesh (TCM) was produced by coating polypropylene with titanium dioxide.^5^ To date, there are very different meshes for LR. TCM have only recently started to be used in the last two decades.^6^ In the literature, there are many studies comparing lightweight and heavyweight meshes. Nevertheless, there is no clear information about the effects of TCM on recurrence, foreign body feeling and chronic pain in LR. The aim of this study was to assess the recurrence, foreign body feeling and chronic pain after LR using TCM.

## METHODS

A single-centre cohort study was designed retrospectively and ethical approval for the study was obtained from Clinical Research Ethics Committee of Ordu University Faculty of Medicine (Date: 7/2/2019, Approval number: 2019-21). Two hundred and fifty-two patients who underwent LR for inguinal hernia were evaluated between May 2014 and January 2018 at the Ordu University Training and Research Hospital in Turkey. Of those patients who used TCM and PM were included in the study. Patients under 18 and over 75 years of age, with a history of femoral hernia, recurrent inguinal hernia, bilateral inguinal hernia, malignancy, ostomy, chemotherapy or radiotherapy, an ASA score of four or above, patients whose information could not be accessed were excluded. The patients were analyzed in terms of primary outcomes (age, gender, body mass index (BMI), smoking habits, comorbid diseases, American Society of Anesthesiologists (ASA) score, hernia type, side of hernia, duration of hernia presentation and operative time) and secondary outcomes (surgical site occurence, recurrence, foreign body feeling and chronic pain). Comorbid diseases included cardiovascular conditions (hypertension, congestive heart failure etc.), respiratory conditions (asthma, chronic obstructive pulmonary disease etc.), diabetes mellitus and chronic renal failure. Surgical site occurrences were defined as surgical site infection (SSI), seroma and hematoma. Operations and postoperative follow-up were performed a single general surgeon. Informed consent about surgery was obtained by the patients before the operation. Patient information was obtained by scanning the medical files or calling the patient on the phone.

TCM (TiO_2_, BioCer™, Germany) was produced by coating polypropylene monofilament with titanium dioxide and had a lightweight (47 g/m^2^) and wide pore size (2.8 mm). Polypropylene mesh (PM) (Proline, Ethicon™, Holland) had a monofilament structure and a highweight (80 g/m^2^) and low pore size (0.8-1.2 mm). Mesh selection was randomized and was not made according to any patient characteristics. Patients were divided into two groups according to the mesh elected (TCM and PM); titanium group (TG) and polypropylene group (PG), respectively.

The types of hernia were classified according to the Nyhus classification.^7^ The long-term outcomes were defined complications as recurrence, foreign body feeling and chronic pain. Outpatient follow-up appointments were conducted at periods of one week, two weeks, one month, three months, six months and one year following the LR. Patients were asked if they had swelling and/or pain in the operative site, and if any of them had, they were then invited back to the hospital. Recurrence was accepted as occur of the hernia which was detected with a physical examination and ultrasound. Patients were asked: “Do you have any foreign body feeling in the operative site?”, the answers by patients were recorded as either a “yes” or a “no”. Patients were asked: “Do you have any pain in the operative site?”, the pain condition of patients was rated from 0 (no pain) to 10 (worst pain imaginable) according to the visual analogue scale (VAS). Chronic pain was defined as pain in the operative site lasting more than 6 months after surgery assessed using the VAS (≥ 3: chronic pain).^8^

All operations were performed using the Lichtenstein tension-free technique under general or spinal anesthesia.^2^ A single-dose of one gm antibiotic (cefazolin sodium) by intravenously was given for prophylaxis 30 minute before the operation. The size of both meshes was 15x10 cm and stitched with a 2/0 polypropylene suture (Prolene, Ethicon™, Amersfoort, Holland).

In this study, the identifiers for continuous variables were: mean, average, standard deviation, minimum and maximum values, and the identifiers for categorical variables were: number and percentage. The data distribution was evaluated using the Kolmogorov-Smirnov test. The Fisher’s exact and chi-square test were used to identify the relationship between categorical variables. The Mann-Whitney U test was used to compare group averages in terms of continuous variables. In calculations, the statistically significant level was determined as 5% (*p*=0.05) and the SPSS (IBM SPSS for Mac, Ver.24) statistics package software was used for calculations.

## RESULTS

In this study, enrollment of 252 patients was completed. Total of 31 patients were excluded as they were lost to follow-up. Two hundred and twenty-one patients; 72 who used TCM and 149 who used PM were included.

### Primary Outcomes

The groups were similar in terms of patient and hernia characteristics (*p>0.05*). Primary outcomes are shown in Table-I. The mean follow-up time was 24.67±7.41 months in TG (min 12-max 39) and 26.05±8.89 months in PG (min 12-max 49). No significant difference was found between groups regarding the follow-up time (*p*=0.331).

**Table-I:** The primary outcomes in patients.

	Titanium group (N=72)	Polypropylene group (N=149)	P value
Age (years)	57.21±14.93(24-75)	55.41±12.77(18-75)	0.951
***Gender***			
Female (%)	67(93.1)	137(91.9)	0.772
Male (%)	5(6.9)	12(8.1)	
BMI (kg/m^2^)	26.58±4.09(18.7-35.3)	28.61±3.83(19.4-36.5)	0.782
Comorbidities			
Cardiovascular (%)	27(37.5)	41(27.5)	
Respiratory (%)	7(9.7)	15(10.1)	
DM (%)	5(6.9)	14(9.4)	0.618
CRF (%)	2(2.8)	3(2)	
***ASA score***			
1 (%)	19(26.4)	34(22.8)	
2 (%)	27(37.5)	85(57)	0.162
3 (%)	26(36.1)	30(20.1)	
Smoking	14(19.4)	18(12.1)	0.145
***Hernia type***			
Nyhus 1	10	16	
Nyhus 2	18	41	0.912
Nyhus 3a	29	59	
Nyhus 3b	15	33	
***Side of hernia***			
Right	38	78	0.729
Left	34	71	
Duration of hernia presentation (months)	40.38±36.19(1-168)	34,69±31.27(1-180)	0.535
Operative time (minute)	38.97±7.47(24-72)	40.21±8.13 (28-66)	0.732

**BMI:** Body mass index, **DM:** Diabetes mellitus, **CRF:** Chronic renal failure, **ASA:** American Society of Anesthesiologists, **SSI:** Surgical site infection.

### Secondary Outcomes

Surgical site occurence was similar in both groups (*p*>0.05). Recurrence was observed in 1.39% (n=1) of TG and 2.01% (n=3) of PG. Foreign body feeling was observed in 15.3% (n=11) of TG and 27.5% (n=41) of PG. Chronic pain was observed in 4.2% (n=3) of TG and 12.8% (n=9) of PG. No significant difference was found in terms of recurrence between groups (*p*>0.05), and foreign body feeling and chronic pain were significantly lower in TG than PG (*p*<0.05). Secondary outcomes were showed in Table-II.

**Table-II:** The secondary outcomes in patients.

	Titanium group (N=72)	Polypropylene group (N=149)	P value
Surgical site occurence			
SSI	10(13.9)	14(9.4)	0.314
Seroma	9(12.5)	19(12.8)	0.958
Hematoma	5(6.9)	9(6)	0.797
Recurrence	1(1.39)	3(2.01)	0.606
Foreign body feeling	11(15.27)	41(27.52)	0.044
Chronic pain	-	6(6.2)	0.047

Percentages are in parenthesis. **SSI:** Surgical site infection.

## DISCUSSION

Inguinal hernia repair is the most common surgery with over 20 million operations performed per year worldwide.^2^ Despite the advantages such as postoperative pain, comfort of laparoscopic hernia repairs, LR is still one of the most common hernia repairs in the world.^9^ Today, there are hundreds of different meshes available on the market. Nevertheless, there is no standard mesh in hernia repair. Additionally, although lightweight meshes cause less foreign body feeling and chronic pain than highweight meshes after open hernia surgery, these results still have heterogeneity.^5^,^10^ In recent years, most studies in the literature were only focused on the use of TCM in laparoscopic hernia surgery. Prassas et al. discovered that the use of TCM in patients who performed totally extraperitoneal inguinal hernia repair did not provide greater benefits than PM in terms of chronic pain and recurrence.^11^ Moreover, in 2019 Yang et al. found that TCM lead to less foreign body feeling than highweight PM in laparoscopic inguinal hernia repair.^12^ Contrary, we focused on the long-term outcomes in LR using TCM in the current study.

SSI after hernia repair is common and associated with many factors such as experience, smoking habits. Pardhan et al. reported a study from Aga Khan University Hospital, Karachi reported the SSI rate of 7.7% in patients undergoing elective surgery including LR and smoking increases this rate.^13^ However, the SSI was higher for all patients (24/221, 10.86%) and no difference was found between smoking and SSI in our study. It is well known that recurrence is one of the complications that can occur following hernia surgery. In a prospective study by Memon et al. in 2017, the recurrence rate was 1.5% after LR. In the present study, this rate was similar in TG and PG (1.39 vs. 2.01, respectively).^14^ Some clinical studies comparing lightweight mesh with heavyweight mesh have previously been identified showing a tendency of less pain with using lightweight mesh in LR.^15^,^16^ In contrast, a randomized and controlled study by Smitenski et al. showed that no significant difference was found between lightweight and highweight meshes in terms of recurrence.^5^ On the other hand, there are studies evaluating TCM in the literature. Example, in a prospective study by Koch et al. found that no significant difference was detected between lightweight TCM and heavyweight PM in patients after 1-year follow-up in terms of recurrence.^6^ This study is very similar to our study with regard to the objective of research and we could not obtain any information such as on pain scoring and time to return to work in the early postoperative period because of the retrospective design of this study. We think that our study is more comprehensive than that of Koch et al.^6^ because we included a longer patient follow-up period and an investigation of the foreign body feeling in our study. In this study, we found similar results to the study of these authors in terms of recurrence.

Materials used in hernia surgery lead to inflammatory response even though they are non-immunogenic and non-toxic. This inflammatory response causes a foreign body reaction in the operative site.^7^ According to recent studies, pores of the meshes with a high pore size are filled with thinner nets than meshes with a low pore size, hence this allows meshes with a higher pore size to be much more flexible than meshes with a lower pore size.^8^,^17^ Post et al. analyzed that most of the patients after the hernia repair with a mesh experienced foreign body feeling, and it was stated that this was caused by the foreign body reaction.^7^ Furthermore, in this study, even though there were no significant differences between patients who had taken lightweight and heavyweight meshes, foreign body feeling found was less in patients with lightweight meshes. Similarly, in an animal experimental study by Scheidbach et al. found that TCM lead to less foreign body reaction than heavyweight meshes.^18^ This result in this study was supportive of our findings, which reveal that foreign body feeling is less in TCM if it is accepted that foreign body feeling is caused by the foreign body reaction. In the literature, there are no studies that examine the effects of TCM on the foreign body feeling in LR. However, we found a significant difference between groups in terms of foreign body feeling. Furthermore, we think that a follow-up period of more than 12 months can reveal a difference between groups in terms of foreign body feeling.

A local inflammatory response caused by meshes used in hernia repair results in scar tissue at the operative site and this also leads to chronic pain.^19^ There is also association between the rate of chronic pain and the type of mesh used for hernia repair.^20^ In 2018, Bona et al. showed that the use of lightweight mesh in LR significantly reduces the incidence of chronic pain.^21^ Similarly, Lee et al. found that the use of lightweight meshes in LR improves functional outcomes and quality of life compared to heavyweight meshes.^22^ However, Carro et al. showed that no significant difference was between lightweight and heavyweight groups in terms of chronic pain.^23^ In the study comparing TCM and PM, Koch et al. showed that chronic pain was less in patients with TCM than in patients with PM.^6^ Similar to previous studies, we observed lower chronic pain using TCM compared to PM.

The ideal mesh is identified as a mesh that is cheap, cost-effective, low-complication, most comfortable and easy to apply, but this has still not been discovered. These are classified according to the composition or type of material as: (1) first-generation (synthetic, non-absorbable-PM), (2) second-generation (mixed or composite prosthesis-TCM), and (3) third-generation (biological).^24^ Additionally, new generation meshes are usually more expensive than previous generation. In the study of Yang et al., PM and TCM were compared in laparoscopic inguinal hernia repair and TCM cost was approximately twice higher than PM (*p<0.001*).^12^ Similarly, the same meshes were compared in the present study. Unfortunately, we could not find an exact cost due to the retrospective design. However, we estimate that the cost was higher in TG. Therefore, we think that surgeons should consider the cost of new generation meshes as well as positive attributes.

### Limitations of the study

Firstly, the numbers of patients were relatively small and retrospective design. Secondly, we could not perform strong and valid tools like the Carolinas Comfort Scale or McGill Pain Scale for postoperative pain and functionality. Despite these limitations, the advantages of this study are that all operations were performed by a single surgeon, that it was a single-centre study, and that only a limited number of studies that investigate recurrence, foreign body feeling and chronic pain in LR are available at all.

## CONCLUSION

In conclusion, in LR, TCM lead to less foreign body feeling and chronic pain than PM, and there is no difference in terms of recurrence between the two meshes. LR is still one of the commonly used inguinal hernia repairs used today with reduction of complications related to the mesh to a minimum among the aims of surgeons. We believe our manuscript will contribute to science on this topic.
